# Effect of Tafamidis on Clinical and Functional Parameters in Transthyretin Amyloid Cardiomyopathy

**DOI:** 10.1016/j.jacadv.2024.101511

**Published:** 2024-12-19

**Authors:** Ting-Wei Kao, Yi-Hsin Hung, An-Li Yu, Mei-Feng Cheng, Mao-Yuan Su, Chi-Chao Chao, Cheng-Hsuan Tsai, Yen-Hung Lin

**Affiliations:** a Department of Internal Medicine, National Taiwan University Hospital, Taipei, Taiwan; b Division of Cardiology, Department of Internal Medicine, National Taiwan University Hospital, Taipei, Taiwan; c Cardiovascular Centre, National Taiwan University Hospital, Taipei, Taiwan; d Department of Nuclear Medicine, National Taiwan University Hospital, Taipei, Taiwan; e Institute of Environmental and Occupational Health Sciences, National Taiwan University, Taipei, Taiwan; f Department of Medical Imaging, National Taiwan University Hospital, Taipei, Taiwan; g Department of Medical Imaging and Radiological Technology, Yuanpei University of Medical Technology, Hsinchu, Taiwan; h Department of Neurology, National Taiwan University Hospital, Taipei, Taiwan; i National Taiwan University College of Medicine, Graduate Institute of Clinical Medicine, Taipei, Taiwan

**Keywords:** amyloidosis, meta-analysis, tafamidis, tissue characterization, transthyretin cardiomyopathy

## Abstract

**Background:**

Transthyretin amyloid cardiomyopathy (ATTR-CM) has recently gained recognition as a rare and fatal disease. Tafamidis, a first-in-class transthyretin stabilizer, has emerged as a promising agent for attenuating disease progression. Nevertheless, how tafamidis alters clinical and imaging parameters remains unclear.

**Objectives:**

This systemic review and meta-analysis aimed to investigate how tafamidis remodels the myocardium and influences the disease trajectory of ATTR-CM.

**Methods:**

PubMed, EMBASE, and the Cochrane Library were searched for literature from inception to February 2024 which reported either the effects of tafamidis treatment or natural course of ATTR-CM. Outcomes of interests were all clinical and imaging parameters available from at least 2 independent studies.

**Results:**

We identified 30 studies comprising 2,973 participants with ATTR-CM. Pooling all studies with outcomes of both tafamidis and placebo, tafamidis significantly reduced all-cause mortality (OR: 0.19; 95% CI: 0.07 to 0.56) and cardiovascular death (OR: 0.08; 95% CI: 0.02-0.30). Tafamidis also ameliorated the deterioration of 6-minute walk distance (standardized mean difference [SMD] 0.04 vs. −0.29, *P* = 0.002) and serum N-terminal pro-B-type natriuretic peptide level (SMD: -0.03 vs 0.41, *P* < 0.001). Regarding imaging parameters, better global longitudinal strain on echocardiography (SMD: 0.06 vs 0.50, *P* = 0.003), heart to contralateral ratio (SMD: −0.23 vs. −1.17, *P* = 0.037) on technetium-99m pyrophosphate scintigraphy, extracellular volume (*P* = 0.003), left (*P <* 0.001) and right (*P* = 0.001) ventricular ejection fraction, and right atrium area (*P* = 0.033) on cardiac magnetic resonance imaging were observed after tafamidis treatment.

**Conclusions:**

Tafamidis improves clinical outcomes and limits the progression of cardiac remodeling in ATTR-CM.

Cardiac amyloidosis is an increasingly recognized and potentially fatal disease. Amyloidosis with cardiac involvement potentiates the development of arrhythmia, reduces myocardial contractility, and eventually leads to adverse cardiovascular events. Among the subtypes, light chain amyloidosis has been known for decades and managed with medications targeting plasma cells.^1^ Recently, transthyretin amyloid cardiomyopathy (ATTR-CM) has been recognized, characterized by the excessive infiltration of misfolded transthyretin protein in the myocardial extracellular space and target organs.^2^ Many potential therapeutic targets have been identified for ATTR-CM, of which the transthyretin stabilizer tafamidis is the first well-established disease-modifying medication. The ATTR-ACT (Safety and Efficacy of Tafamidis in Patients With Transthyretin Cardiomyopathy) trial ^3^ demonstrated that tafamidis was a first-in-class medication that improved all-cause mortality, cardiovascular-related hospitalizations, functional decline, and quality of life. Till now, tafamidis is the only new agent approved for ATTR-CM on both hereditary and wild-types.

The better clinical endpoints in patients with ATTR-CM who receive tafamidis treatment have been attributed to attenuated cardiac remodeling by amyloid deposition. Various imaging modalities have been applied to assess anatomical, structural, and functional changes. Echocardiography has been traditionally used to assess cardiac structure and function. However, traditional 2-dimensional echocardiographic parameters may be suboptimal for monitoring the disease course of ATTR-CM due to the subtle changes that occur during its progression.^4^ Speckle tracking echocardiography can provide additional information as it assesses the degree and regional pattern of compromised global longitudinal strain (GLS), and it has been shown to be a good screening and prognostic tool for ATTR-CM.^5^ Regarding other modalities, cardiac magnetic resonance imaging (CMR) is used to evaluate amyloid infiltration and interstitial fibrosis via late gadolinium enhancement, native T1 mapping and extracellular volume (ECV),^6^ and technetium-99m scintigraphy is used to quantify radiotracer uptake in the myocardium by planar imaging, therein illustrating regional amyloid accumulation.^7^ Nevertheless, CMR and cardiac scintigraphy are still not typically repeated in clinical practice to monitor disease activity.

Although many studies have focused on the effects of tafamidis on patients with ATTR-CM, a universally standardized protocol to monitor treatment response has not yet been established. In addition, small sample sizes and inconsistent use of parameters have been prominent limitations of these studies. Even though ATTR-ACT has provided beneficial evidence in hard outcomes, the efficacies of tafamidis on other imaging outcomes remained unknown. Consequently, further research is needed to evaluate the efficacy of tafamidis on cardiac remodeling compared to the natural course of ATTR-CM.^8^ The aim of this study was therefore to systemically elucidate how tafamidis treatment alters cardiac tissue characterization and translates to clinical manifestations in patients with ATTR-CM.

## Methods

The systematic review and meta-analysis was registered in an international prospective register of systematic reviews (PROSPERO), with a certification number of CRD42023430879. The study adhered to the principles of the PRISMA (Preferred Reporting Items for Systematic Reviews and Meta-Analyses) guidelines. Meta-analysis involves the collection of existing data and therefore approval of Institutional Review Board is exempt.

### Search strategy

PubMed/MEDLINE, Embase, and the Cochrane Library were searched from inception to February 2024. Published literature was reviewed without language restriction. Only human studies were considered, and presented conference abstracts were not included due to limited data sets. The literature search was based on the following medical subject headings and relevant keywords: cardiac amyloidosis, ATTR, transthyretin, echocardiography, magnetic resonance imaging, bone scintigraphy, and technetium-99m pyrophosphate (Supplemental Table 1). Both randomized controlled trials (RCTs) and observational cohort studies were considered for inclusion if they fulfilled the following criteria: 1) enrolled individuals with confirmed ATTR-CM; 2) assessed the effect of tafamidis, placebo, or no treatment; and 3) had available data on clinical and/or imaging parameters both before and after treatment. The exclusion criteria were: 1) case reports in which only one patient was described; 2) studies which published the outcomes only of medical treatment other than tafamidis; and 3) patients who concurrently receives other disease-modifying medications aside from tafamidis.

### Outcomes

Endpoints were defined as any clinical or imaging parameter of echocardiography, technetium-99m scintigraphy, and CMR reported in at least 2 separate studies. Clinical parameters included cardiovascular events such as all-cause mortality, cardiovascular death, and a composite of cardiovascular event hospitalization, heart failure hospitalization, or heart failure exacerbation, as well as aerobic endurance indicated by 6-minute walk distance (6MWD) and serum level of N-terminal pro-B-type natriuretic peptide (NTproBNP). Echocardiographic parameters referred to anatomical and functional assessments as recommended by the American Society of Echocardiography and European Association of Cardiovascular Imaging.^9^ Technetium-99m scintigraphy was used for volumetric evaluation based on radioactivity in the heart divided by that in the contralateral lung and presented as heart-to-contralateral (H/CL) ratio. The tracer of technetium-99m scintigraphy included pyrophosphate, 3,3-diphosphono-1,2-propanodicarboxylicacid, and hydroxymethylene diphosphonate. CMR was used to evaluate amyloid burden according to the ECV derived from native and post-contrast T1 mapping based on region-of-interest methodology.^10^

### Data extraction

The titles and abstracts of the identified articles were preliminarily screened by 3 authors (T.W.K., Y.H.H., and C.H.T.), and subsequently the full texts were reviewed by all study investigators before final inclusion. Disputes at any step were resolved by consensus. Data on the study design (eg, randomized trial or cohort study), baseline characteristics of the cohort (eg, age, sex, follow-up duration, etc.), and outcomes before and after treatment were extracted. The number of events and sample sizes were extracted for the clinical cardiovascular events (ie, all-cause mortality, cardiovascular death, and heart failure hospitalization). Continuous variables were retrieved including mean ± SD, median (IQR), median (95% CI), least squares means with standard error of mean, or 95% CI and ratio of adjusted geometric means with 95% CI. Finally, these trials reported continuous outcome metrics in various forms (eg, median, least-square mean difference, and ratio of adjusted geometric mean difference) without raw data, such as mean and standard deviation. Consequently, statistical approaches varied, and individual trial outcomes (significant or insignificant) may change once transformed to standardized mean difference (SMD) for this meta-analysis. Nonetheless, the pooled estimate remains meaningful, as it effectively consolidates the available evidence, despite potential inaccuracies. We extracted information from the studies that provided both pre-treatment and post-treatment data.

If 2 or more separate studies shared the same enrolled cohort and outcomes, we chose the one with the longer follow-up period. If these studies used the same follow-up duration, the earlier published study was enrolled. We enrolled all studies with the same included population but with different outcomes, and used the earliest published study as the primary and others as supplementary data.

### Statistical analysis

The meta-analysis was conducted using Comprehensive Meta-Analysis, Version 3. Every imaging parameter that has been reported in at least 2 different studies was analyzed. A random effects model based on the DerSimonian and Laird method was applied. For the categorical outcomes (clinical events), we obtained the effect size (OR) from a table containing the number of subjects and the number of events. When analyzing continuous outcome data, the meta-analysis comprised 2 types of analyses. First, the outcomes of tafamidis and placebo arms were summarized separately, and comparisons between arms were made using subgroup analysis (with the mixed effects model); this approach was termed indirect comparison. Notably, the placebo arm was extracted from studies comparing any agents (ie, tafamidis, patisiran, and inotersen) to a placebo, or from studies that exclusively included a control group. Second, only studies which directly compared tafamidis to placebo arms were included in the meta-analysis. The available number of studies was expected to be smaller in the second approach, which we termed direct comparison. Due to the varied sources of data metrics, we transformed the continuous data into SMD for subsequent pooled meta-analysis. ORs with 95% CIs were assessed, and a 2-tailed *P* value <0.05 indicated statistical significance.

### Quality of evidence and risk of bias

The risk of bias of the included RCTs and cohort studies was evaluated using the Cochrane risk of bias tool and ROBINS-I (Risk Of Bias In Non-randomized Studies of Interventions), respectively (Supplemental Table 2).

## Results

### Search results

**Figure 1:**
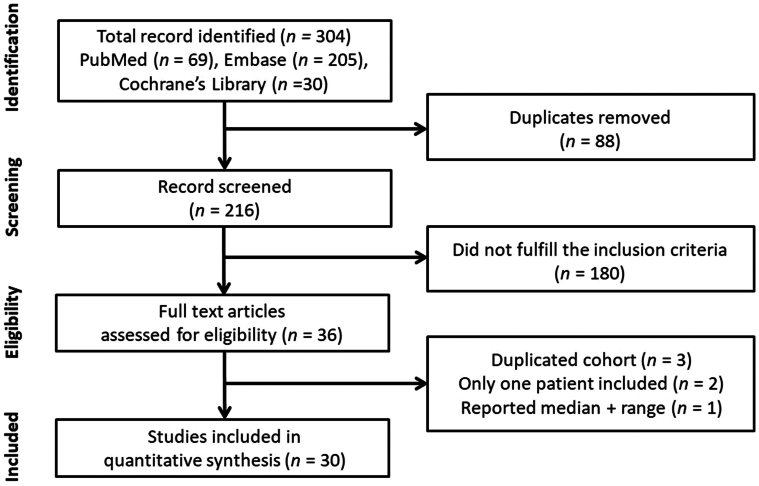
Literature Search Preferred Reporting Items for Systematic Reviews and Meta-Analysis (PRISMA) flow diagram illustrated the process for study inclusion.

A total of 304 articles were identified in the initial search, of which 88 duplicate studies were removed. A further 180 studies were excluded because of irrelevant topics after examining the abstracts. After reviewing the full texts of the remaining 36 manuscripts, 6 articles were removed due to the reasons shown in Figure 1. Finally, 30 studies were included for meta-analysis (Figure 1).^3^^,^^11^, ^12^, ^13^, ^14^, ^15^, ^16^, ^17^, ^18^, ^19^, ^20^, ^21^, ^22^, ^23^, ^24^, ^25^, ^26^, ^27^, ^28^, ^29^, ^30^, ^31^, ^32^, ^33^, ^34^, ^35^, ^36^, ^37^, ^38^, ^39^

### Study characteristics

Among the included studies, there were 7 clinical trials or post hoc subgroup analyses (3 related to the ATTR-ACT trial and 2 to the APOLLO trial). The remaining studies were observational cohort studies or case series. A total of 2,973 participants were included, of whom 1,624 (54.6%) received tafamidis treatment. Fifteen studies reported both the outcomes of patients receiving tafamidis and those who received no treatment as placebo. Ten studies reported the efficacy of disease-modifying treatment, and 5 studies only reported the natural course of ATTR-CM. The enrolled subjects were older and predominantly male. The 6MWD at baseline ranged from low-normal to mildly reduced, while serum levels of NTproBNP were remarkably elevated. Details of the baseline demographics and clinical characteristics are summarized in Table 1.

**Table 1:** Baseline Demographic and Clinical Characteristics

First Author, Year	Study Design	Direct Comparison	Treatment	N	Follow-Up	Age, y	Male	6MWD (m)	NTproBNP (pg/mL)	LVEF^a^
Falk, 2011^11^	Cohort study	Yes	Tafamidis	35	12 mo	NA	91%	NA	NA	NA
			None	29	12 mo	NA	NA	NA	NA	NA
Merlini, 2013^12^	RCT	No	Tafamidis	21	12 mo	63.1	61.9%	NA	NA	50.3%
Maurer, 2018^3^	RCT	Yes	Tafamidis	264	30 mo	74.5	91.3%	350.6	2,996	NA
			Placebo	177	30 mo	74.1	88.7%	353.3	3,161	NA
Solomon, 2019^13^	RCT	No	Placebo	36	18 mo	62	83.3%	NA	845.7	62.2%
Fontana, 2021^14^	Cohort study	No	Placebo	16	12 mo	69	87.5%	463	1,659	NA
Miller, 2021^15^	Cohort study	No	Same as Maurer, 2018							
Rettl, 2021^16^	Cohort study	Yes	Tafamidis (1)^c^	64	9-12.5 mo	NA	NA	377.1	2,633	NA
			Tafamidis (2)^c^	23	9-12.5 mo	NA	NA	415.4	2,420	NA
			None	54	9-12.5 mo	NA	NA	388.1	2,798	NA
Doumas, 2022^17^	Cohort study	No	Tafamidis	5	12 mo	76.2	100%	419	1,559.2	52.4%
Elsadany, 2022^18^	Cohort study	No	Tafamidis	5	12.5 mo	73.8	100%	NA	NA	NA
Giblin, 2022^19^	Cohort study	No	Tafamidis	23	12 mo	79.13	100%	NA	2,607	45.8%
			None	22	12 mo	78.24	91%	NA	2,807	48%
Ochi, 2022^20^	Cohort study	No	Tafamidis	38	16.4 mo	78.4	87%	NA	231	50%
			None	44	16.8 mo	84.3	86%	NA	281	51%
Odouard, 2022^21^	Cohort study	No	Tafamidis	52	12 mo	77	81%	NA	NA	NA
			No treatment	8	12 mo	NA	NA	NA	NA	NA
Rettl, 2022^22^	Cohort study	Yes	Tafamidis (1)^c^	35	9 mo	78.3	82.9%	397.9	2,265.5	50.0%
			Tafamidis (2)^c^	15	11 mo	74.9	86.7%	412.4	2,292.0	50.6%
			None	19	NA	78.4	78.9%	404.0	2,664.5	48.4%
Chamling, 2023^23^	Cohort study	Yes	Tafamidis	20	12 mo	76	90%	NA	2,068	NA
			None	20	12 mo	80	75%	NA	1,810	NA
Garcia-Pavia, 2023^24^	RCT	No	Placebo	13	12 mo	68	100%	418	1,591	72%
Ghoneem, 2023^25^	Cohort study	Yes	Tafamidis	421	12 mo	76.8	85.7%	NA	1,365.3	44.8%
			None	421	12 mo	76.2	86.7%	NA	1,015.8	46.0%
Ichikawa, 2023^26^	Cohort study	No	Tafamidis	41	16 mo	75.6	90.2%	NA	271.9^b^	52.9%
Kim, 2023^27^	Cohort study	Yes	Tafamidis	79	1.7 y	NA	NA	NA	NA	NA
			None	35	1.7 y	NA	NA	NA	NA	NA
Lee, 2023^28^	Cohort study	Yes	Tafamidis	3	1,043 d	62.3	100%	NA	NA	NA
			None	2	462 d	62	100%	NA	NA	NA
Maurer, 2023^29^	RCT	No	Placebo	179	30 mo	76	90%	367.7	1,813.0	NA
Nakaya, 2023^30^	Cohort study	No	Tafamidis	8	12 mo	77.0	87.5%	NA	2,695	59.2%
Papathanasiou, 2023^31^	Cohort study	No	Tafamidis	14	9 mo	76.4	78.57%	NA	4,872	53%
Rettl, 2023^32^	Cohort study	No	Tafamidis	40	9 mo	78.6	82.5	NA	2,181	49.1%
Rosenblum, 2023^33^	RCT	No	Placebo	36	18 mo	63.4	87.5%	NA	845.7	60%
Shah, 2023^34^	RCT	No	Tafamidis	176	30 mo	75.2	89.8%	344.8	3,122	48.0%
			Placebo	177	30 mo	74.1	88.7%	353.3	3,161	48.6%
Takashio, 2023^35^	Cohort study	Yes	Tafamidis	125	12 mo	75.6	88%	NA	187	50.3%
			None	55	12 mo	78.9	87%	NA	329	52.3%
Tsai, 2023^36^	Cohort study	No	Tafamidis	14	12 mo	62.1	93%	NA	2,078.1	56.6%
Wu, 2023^37^	Cohort study	No	Tafamidis	20	12 mo	62.4	80%	NA	1,725	59.2%
Yu, 2023^38^	Cohort study	Yes	Tafamidis	21	599 d	63	71%	NA	511	NA
			None	6	534 d	63	67%	NA	348	NA
Ney, 2024^39^	Cohort study	No	Tafamidis	62	6 mo	79	88.7%	2,177	NA	NA

6MWD = 6-minute walk distance; LVEF = left ventricular ejection rate; N/A = not available; NTproBNP = N-terminal pro-B-type natriuretic peptide; RCT = randomized controlled trial.

a Assessed by echocardiogram.

b BNP.

c Different doses: (1) 61 mg/d, (2) 20 mg/d.

### Echocardiography and speckle tracking analysis

**Figure 2:**
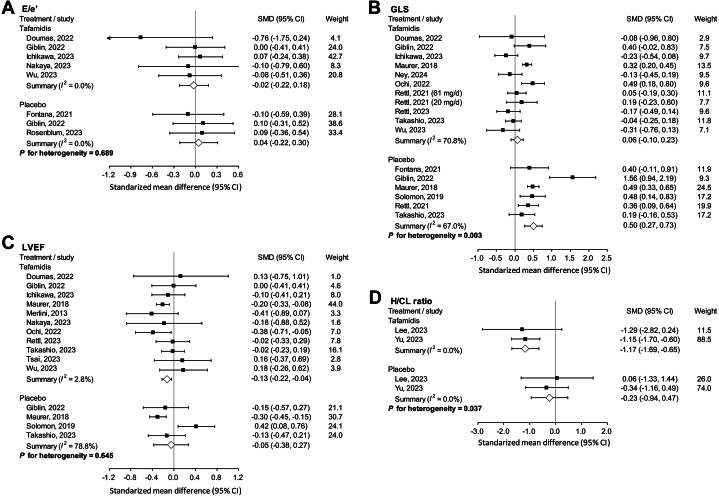
Echocardiographic and Technetium-99m Scintigraphic Parameters of Studies Which Pooled the Data of Tafamidis and Placebo Separately Echocardiographic and technetium-99m scintigraphic parameters of studies which pooled the data of tafamidis and placebo separately, including (A) E/e’; (B) global LS; (C) LVEF; (D) H/CL ratio. LS = longitudinal strain; LVEF = left ventricular ejection fraction; H/CL = heart to contralateral.

Regarding diastolic function, the change in E/e’ was unremarkable in both those who received tafamidis^17^^,^^19^^,^^26^^,^^30^^,^^37^ and those who did not receive disease-modifying treatment^14^^,^^19^^,^^33^ (*P* for interaction = 0.689) (Figure 2A). Regarding systolic capacity, the deterioration in GLS was significantly ameliorated by tafamidis^16^^,^^17^^,^^19^^,^^20^^,^^26^^,^^29^^,^^32^^,^^35^^,^^37^^,^^39^ compared with the natural disease course^3^^,^^13^^,^^14^^,^^16^^,^^19^^,^^35^ (SMD 0.06 vs 0.50, *P* for interaction = 0.003) (Figure 2B). Nevertheless, tafamidis did not significantly affect left ventricular ejection fraction (LVEF)^3^^,^^12^^,^^17^^,^^19^^,^^20^^,^^26^^,^^30^^,^^32^^,^^35^, ^36^, ^37^ (*P* for interaction = 0.645) (Figure 2C). Other outcomes of echocardiography and speckle tracking are summarized in Supplemental Figures 1 and 2, respectively.

### Technetium-99m scintigraphy

H/CL was the most commonly reported quantifiable scintigraphy parameter, and data were available in 2 separate studies. In these 2 studies, pyrophosphate was used as the radiopharmaceutical agent. There was no significant change in H/CL ratio in the control group^28^^,^^38^ (SMD: −0.23; 95% CI: −0.94-0.47), while it significantly decreased in the tafamidis group^28^^,^^38^ (SMD: −1.17; 95% CI: −1.69 to −0.65). The mixed effects model showed a significant interaction effect (*P* for interaction = 0.037) (Figure 2D).

### Cardiac magnetic resonance imaging

**Figure 3:**
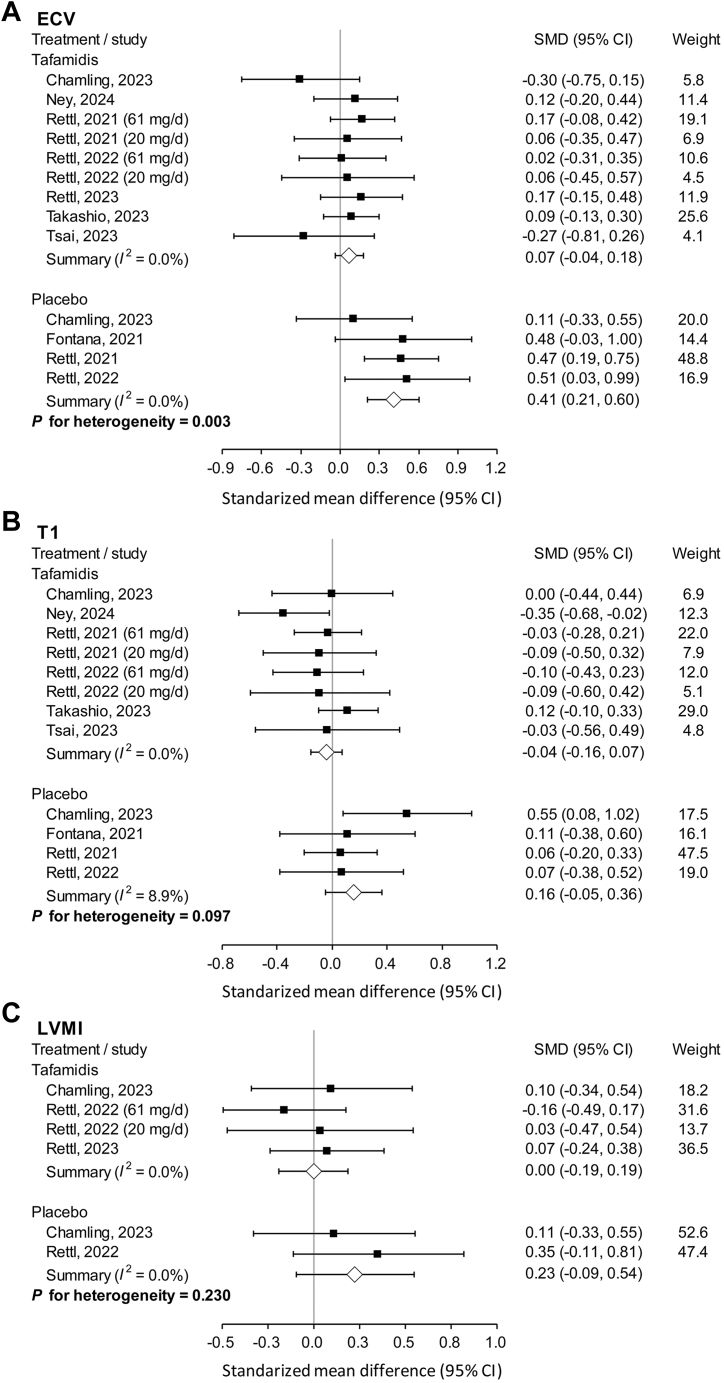
Amyloid Content by CMR of Studies Which Pooled the Data of Tafamidis and Placebo Separately Amyloid content by cardiac magnetic resonance imaging of studies which pooled the data of tafamidis and placebo separately, including (A) ECV; (B) native T1 mapping; (C) LVMI. ECV = extracellular volume; LVMI = left ventricular mass index.

Amyloid burden was assessed by CMR. There was no significant change in ECV in those who received tafamidis treatment^16^^,^^22^^,^^23^^,^^35^^,^^36^^,^^39^ (SMD: 0.07; 95% CI: −0.04-0.18), whereas ECV significantly increased in the placebo arm^14^^,^^16^^,^^22^^,^^23^ (SMD: 0.41; 95% CI: 0.21-0.60), with a significant interaction (*P* for interaction = 0.033) (Figure 3A). However, changes in native T1 mapping^14^^,^^16^^,^^22^^,^^23^^,^^35^^,^^36^^,^^39^ (*P* for interaction = 0.097) (Figure 3B) and left ventricular mass index^22^^,^^23^^,^^32^ (*P* for interaction = 0.230) (Figure 3C) were comparable between treatment and placebo groups.

**Figure 4:**
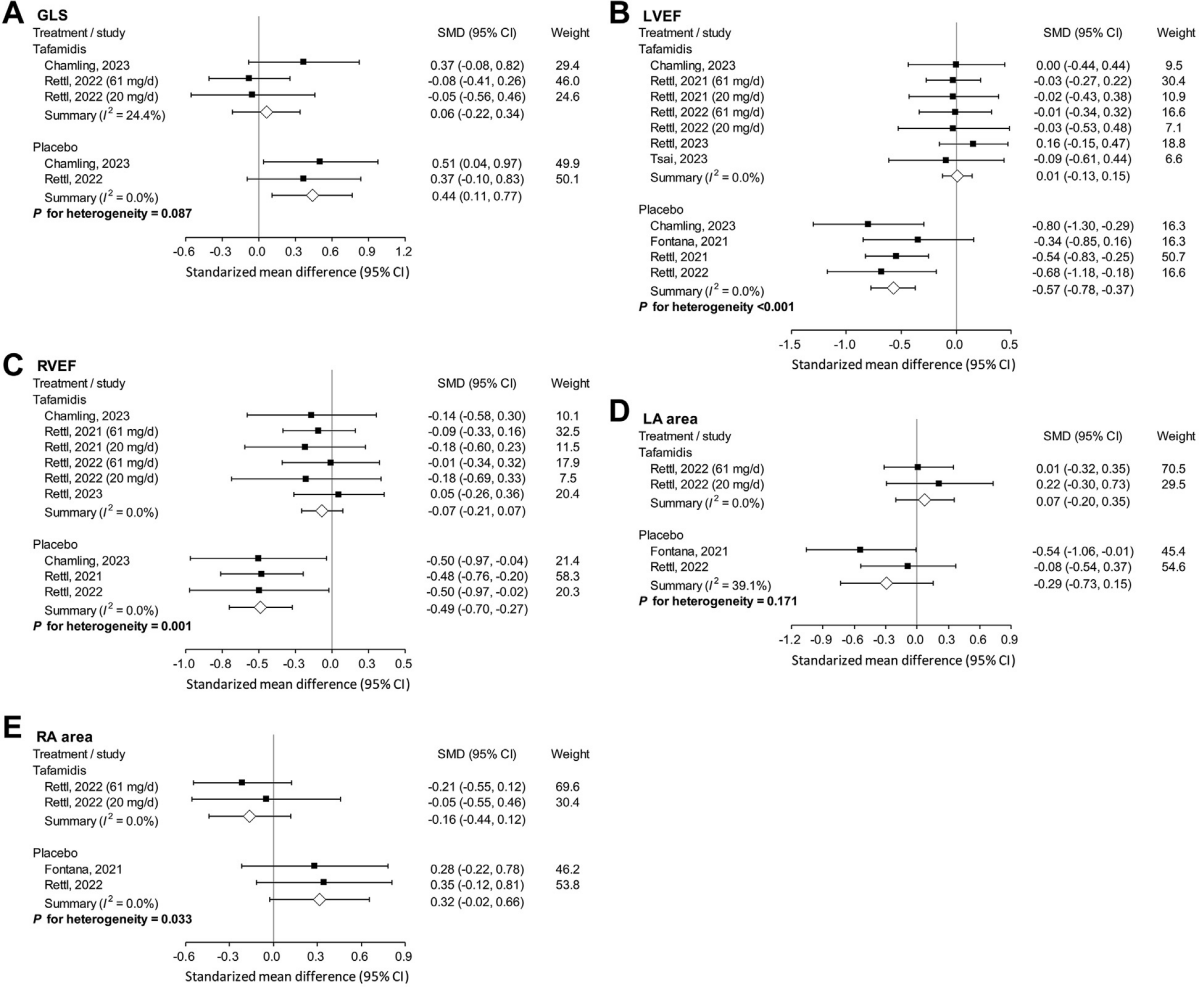
Functional Assessment by CMR of Studies Which Pooled the Data of Tafamidis and Placebo Separately Functional assessment by cardiac magnetic resonance imaging of studies which pooled the data of tafamidis and placebo separately, including (A) global LS; (B) LVEF; (C) RVEF; (D) LA area; (E) RA area. CMR = cardiac magnetic resonance imaging; LA = left atrium; RA = right atrium; RVEF = right ventricular ejection fraction; other abbreviations as in Figure 2.

Regarding functional variables, there was no significant difference in GLS in those treated with tafamidis, but it was aggravated in those who did not receive disease-modifying treatment. The difference, however, did not reach statistical significance^22^^,^^23^ (*P* for interaction = 0.087) (Figure 4A). LVEF^14^^,^^16^^,^^22^^,^^23^^,^^32^^,^^36^ (*P* for interaction <0.001) (Figure 4B) and right ventricular ejection fraction (RVEF)^16^^,^^22^^,^^23^^,^^32^ (*P* for interaction = 0.001) (Figure 4C) remained stable in those who received tafamidis but notably decreased in the placebo arm. Anatomically, there was no significant difference in left atrium area in both tafamidis and placebo arms^14^^,^^22^ (*P* for interaction = 0.171) (Figure 4D). Nevertheless, the right atrium area was maintained by tafamidis (SMD -0.16; 95% CI: −0.44 to 0.12), but tended toward enlargement if left untreated^14^^,^^22^ (SMD 0.32; 95% CI: −0.02 to 0.66), with a significant interaction (*P* for interaction = 0.033) (Figure 4E). Other outcomes of CMR are listed in Supplemental Figure 3.

### Clinical outcomes

**Figure 5:**
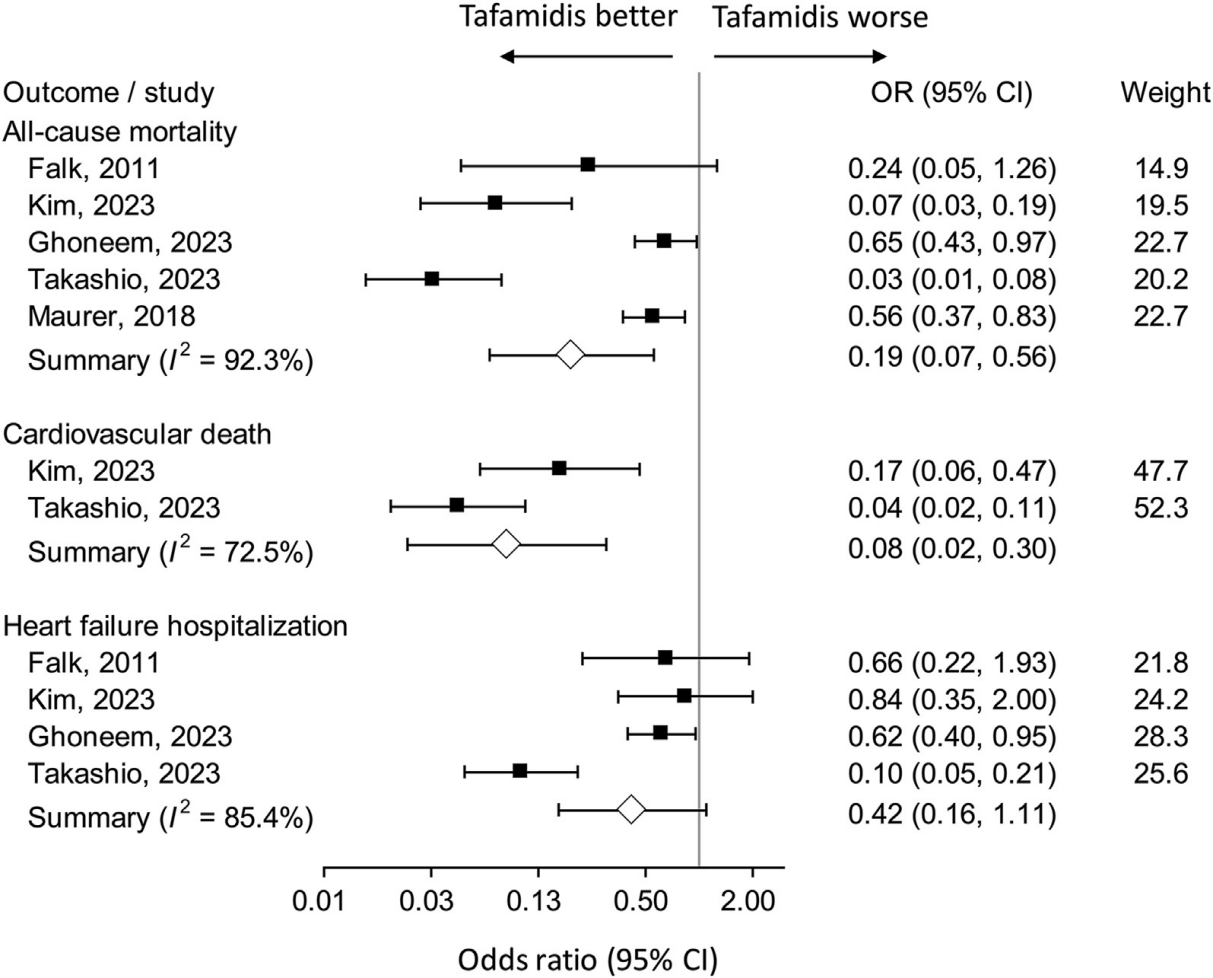
**Major Adverse Cardiac Events by Pooling Studies Which Directly Compared Tafamidis to Placebo** All-cause mortality; cardiovascular death; composite cardiovascular event hospitalization, heart failure hospitalization, or heart failure exacerbation.

Cardiac adverse events were analyzed by including the studies which directly compared tafamidis and control groups. The results demonstrated that tafamidis was significantly associated with a reduced risk of all-cause mortality^3^^,^^11^^,^^25^^,^^27^^,^^35^ (OR: 0.19; 95% CI: 0.07-0.56; *I*^2^ = 92.3%) and cardiovascular death^27^^,^^35^ (OR: 0.08; 95% CI: 0.02-0.30, *I*^2^ = 72.5%). However, composite hospitalizations^11^^,^^25^^,^^27^^,^^35^ due to cardiovascular events, heart failure, or heart failure exacerbations were not reduced by tafamidis compared with placebo, although there was a trend toward significance (Figure 5). Notably, since high heterogeneity was present among the studies due to different patient characteristics and follow-up durations, the pooled results of the meta-analysis were highly heterogeneous.

**Figure 6:**
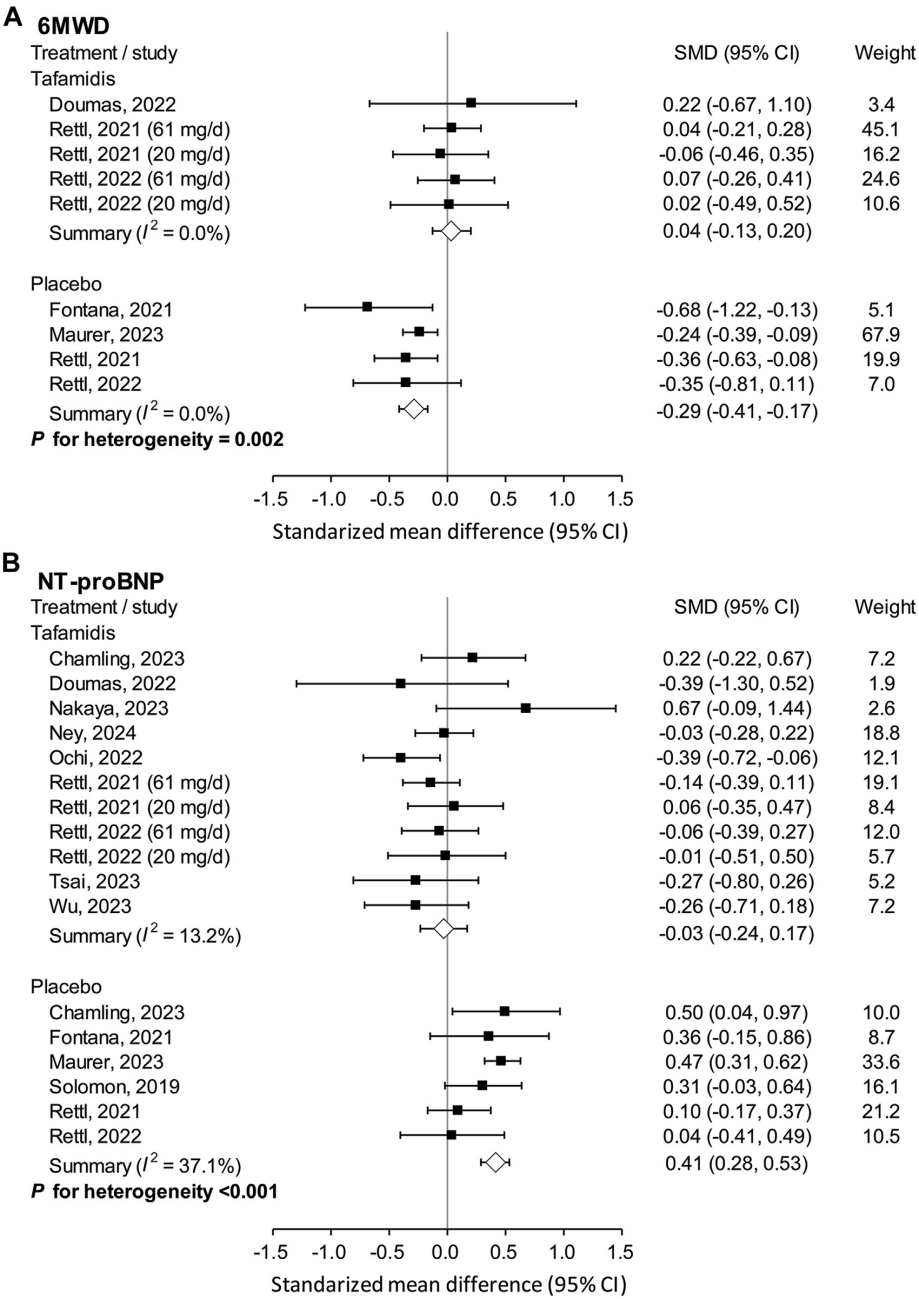
Clinical Outcomes of Studies Which Pooled the Data of Tafamidis and Placebo Separately Clinical outcomes of studies which pooled the data of tafamidis and placebo separately, including (A) 6MWD; (B) serum level of NTproBNP. 6MWD = 6-minute walk distance; NTproBNP = N-Terminal pro-B-type natriuretic peptide.

In the patients who received tafamidis, there was no significant difference in 6MWD before and after treatment^16^^,^^17^^,^^22^ (SMD: 0.04; 95% CI: −0.13 to 0.20), whereas the 6MWD significantly deteriorated in those who did not receive disease-modifying treatment^16^^,^^22^^,^^29^^,^^35^ (SMD: −0.29; 95% CI: −0.41 to −0.71) (Figure 6A). Similarly, serum levels of NTproBNP remained stable in those who received tafamidis treatment^16^^,^^17^^,^^20^^,^^22^^,^^23^^,^^30^^,^^36^^,^^37^^,^^39^, but were remarkably elevated in those who did not receive treatment^13^^,^^14^^,^^16^^,^^22^^,^^23^ (tafamidis: SMD: -0.03; 95% CI: −0.24 to 0.17; control: SMD 0.41; 95% CI: 0.28-0.53) (Figure 6B). The mixed effects models showed significant interactions (*P* = 0.002 for 6MWD and *P* < 0.001 for NTproBNP), indicating that the pooled outcomes (the change from pre-treatment to post-treatment) significantly differed between the 2 groups.

### Sensitivity analysis

**Figure 7:**
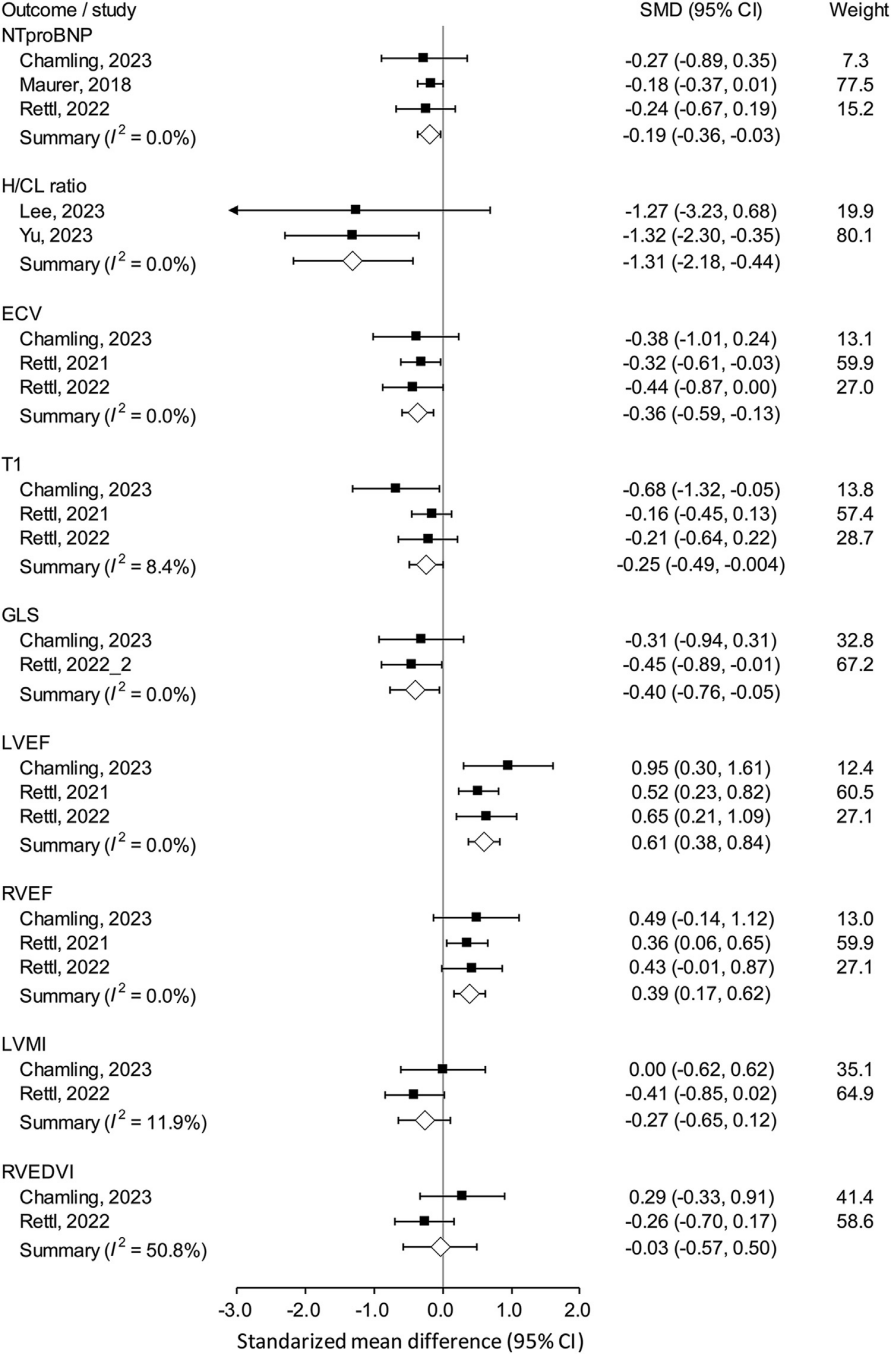
Echocardiographic of Studies Which Directly Compared Tafamidis to Placebo Arms Echocardiographic of studies which directly compared tafamidis to placebo arms, including NTproBNP; LVEF; H/CL; ECV; native T1 mapping; global LS; RVEF; LVMI; RVEDVI. RVEDVI = right ventricular end-diastolic volume index; other abbreviations as in Figures 2, 3, 4, and 6.

To confirm the results, we pooled all studies which reported both tafamidis treatment and placebo or no treatment arms as sensitivity analysis. The results demonstrated greater improvements from pre-treatment to post-treatment in NTproBNP level^3^^,^^22^^,^^23^, H/CL ratio^28^^,^^38^, ECV^16^^,^^22^^,^^23^, T1^16^^,^^22^^,^^23^, GLS^22^^,^^23^, LVEF^16^^,^^22^^,^^23^, and RVEF^16^^,^^22^^,^^23^ in the tafamidis group, with low heterogeneity (*I*^2^ < 10%; Figure 7). However, the effect of tafamidis on left ventricular mass index ^22^^,^^23^ and right ventricular end-diastolic volume index ^22^^,^^23^ was not observed in the meta-analysis results using the direct comparison approach, mainly due to the limited number of included studies (Central Illustration).

**Central Illustration:**
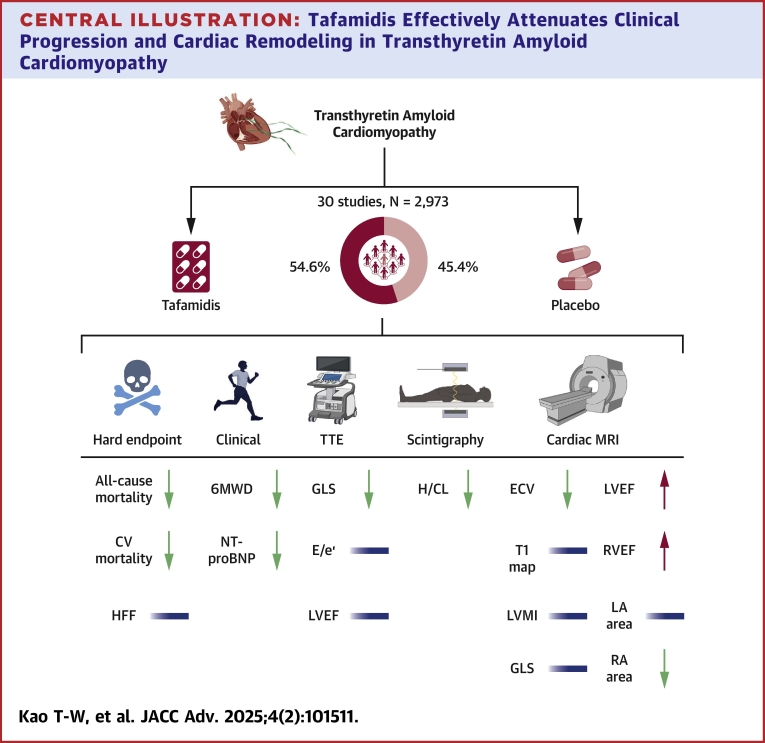
Tafamidis Effectively Attenuates Clinical Progression and Cardiac Remodeling in Transthyretin Amyloid Cardiomyopathy HFF = heart failure hospitalization; TTE = trans-thoracic echocardiogram; other abbreviations as in Figures 2, 3, 4, and 6.

Besides, few included randomized trials provided echocardiography parameters, CMR, and technetium-99m scintigraphy. We therefore conducted a subgroup analysis on all-cause mortality^3^^,^^11^^,^^25^^,^^27^^,^^35^ and NT-proBNP^3^^,^^22^^,^^23^ to compare results between analyses that included and excluded RCTs (Supplemental Figure 4). The nonsignificant interaction effects (0.117 for mortality and 0.725 for NT-proBNP) indicated that the results were not substantially different between observational studies and randomized trails. At last, subgroup analysis of follow-up duration (≤12 vs >12 months) was also conducted, showing no significant difference in pooled estimates between studies with shorter versus longer follow-up (Supplemental Table 3). Additionally, due to an insufficient number of studies, outcomes such as mean age, proportion of male participants, and LVEF could not be estimated in the meta-regression.

## Discussion

To the best of our knowledge, this is the first meta-analysis to explore the impact of tafamidis imaging parameters on ATTR-CM. Beyond cardiovascular outcomes, the results demonstrated anatomical and functional remodeling of the myocardium following tafamidis treatment, as evidenced by cardiac tissue characterization. The major findings of the study were that tafamidis: 1) substantially reduced all-cause mortality and cardiovascular mortality; 2) improved functional capacity as indicated by 6MWD; 3) decreased serum NTproBNP level; 4) stabilized the accumulation of amyloid deposition; and 5) ameliorated myocardial functional remodeling associated with ATTR-CM.

The clinical trajectory of ATTR-CM is characterized by progressive deterioration and is associated with poor cardiovascular outcomes. Infiltrating misfolded transthyretin stiffens and disrupts cardiac structure, thereby compromising cardiac diastolic function and inducing cardiac arrhythmia.^40^ A previous epidemiological meta-analysis identified a high prevalence of cardiac amyloidosis, especially in patients with heart failure with preserved ejection fraction and in older patients with aortic stenosis.^41^ Recently introduced novel disease-modifying therapies have been shown to slow the pathogenesis of ATTR-CM, and tafamidis has been shown to be effective in stabilizing transthyretin accumulation and consequently improving long-term clinical outcomes. The ATTR-ACT randomized control trial demonstrated that tafamidis improved clinical parameters compared to placebo, especially in the early stage of the disease.^3^ In the current study, we pooled all patients across disease severity and found that tafamidis improved many key clinical outcomes. However, due to the limited number of studies and data, the therapeutic effects of tafamidis in patients with different severity of ATTR-CM could not be investigated in this study. Further investigations are warranted to investigate the effects of tafamidis with respect to different disease stages.

Regarding imaging parameters, although only small cohort studies and case series are currently available for analysis, they are emerging as surrogate markers to reflect disease progression and monitor therapeutic responses. Changes in follow-up imaging results in patients with ATTR-CM after tafamidis treatment have attracted increasing attention; however, the modalities and protocols used among previous studies have varied and been inconsistent. This meta-analysis comprehensively investigated the effects of tafamidis on amyloid deposition, as well as cardiac structure and function. ECV was shown to be an independent predictive factor for mortality, and it was also shown to be correlated with amyloid content in patients with ATTR-CM.^42^ Two pioneering studies applying CMR demonstrated that tafamidis treatment could stabilize ECV progression in patients with wild-type ATTR.^22^^,^^23^ Our previous cohort study further validated that tafamidis treatment was associated with decreased ECV in patients with hereditary ATTR-CM.^34^ In addition, ECV has been well correlated with tracer uptake in technetium-99m scintigraphy,^40^ and the results of the present study showed a marked reduction in H/CL ratio after tafamidis treatment. Native T1 mapping directly measures intrinsic signals from the myocardium, encompassing both intracellular and extracellular spaces. Unlike T1 mapping, ECV specifically reflects alterations in the extracellular space. As a result, ECV measurements can accurately assess the extent of extracellular space infiltration, making it a more precise marker of infiltration and enabling its use as a tool for predicting outcomes and monitoring treatment response.^43^^,^^44^ In the current meta-analysis, we did not find differences after tafamidis treatment in native T1 mapping, which may be due to differences in field strengths, techniques of acquisition, and vendors among studies.^45^ Differences in left ventricular mass index, which is a less sensitive marker of amyloid infiltration, as quantified by CMR were also not significant.

Regarding functional assessments, none of the transthoracic echocardiographic parameters exhibited statistically significant alterations after tafamidis treatment. Substantial differences in both LVEF and RVEF were present on CMR, which is compatible with literature suggesting that amyloid deposition leads to impaired systolic capacity and heart failure. Moreover, GLS has been shown to not only be a sensitive biomarker of cardiac function but also long-term survival in patients with ATTR and light chain amyloidosis.^46^ Changes in GLS after tafamidis treatment were only significant in speckle tracking echocardiography but not CMR, although these 2 modalities have previously exhibited fair agreement in left ventricular functional measurements.^47^ These discrepancies may be attributable to differences in disease severity of the included cohorts as well as reporting bias. Anatomical alterations, however, were not evident. This may be because the limited follow-up durations of the included studies were not long enough to reveal the beneficial effects of tafamidis.

Our results suggest that tafamidis may not only improve key clinical outcomes but also help stabilize myocardial remodeling, as indicated by the selected imaging parameters. Although a previous meta-analysis also found that tafamidis could improve survival, few studies have investigated the effect of disease-modifying treatments on anatomical and functional alterations of the myocardium.^48^ By analyzing a range of parameters from various imaging modalities, we provide valuable evidence on tissue characterization following tafamidis treatment, compared to the natural progression of ATTR-CM. In addition, the results remained consistent in head-to-head comparisons between treatment with disease-modifying medications and placebo, facilitating future investigations on how the amelioration of myocardial infiltration of amyloid fibrils may translate to clinical improvements.

### Study limitations

First, patients with both hereditary and wild-type ATTR-CM were pooled for meta-analysis. Since several studies reported outcomes by pooling individuals with different genotypes, we were unable to differentiate the impacts between hereditary and wild-type ATTR-CM on the therapeutic effects of tafamidis. Second, the imaging modalities and parameters reported varied significantly across studies. The meta-analysis was limited to the commonly documented items. Besides, limited imaging results was reported by RCT, mandating future studies to further validate the effect of tafamidis on imaging characteristics. Further standardization of protocols for monitoring treatment response in individuals with ATTR-CM is warranted as well. Third, the reporting of 6MWD typically requires a standardized protocol to ensure consistent quality. In this meta-analysis, the lack of detailed numerical data for 6MWD from the ATTR-ACT trial limits the comprehensiveness of our analysis. However, the consistency between our pooled meta-analysis results and the findings from the ATTR-ACT trial regarding the efficacy of tafamidis was observed. Fourth, given that tafamidis has only received approval for ATTR-CM in recent years, the longest follow-up period in the included studies was no more than 2 years. The durability of the therapeutic efficacy of tafamidis remains uncertain. Fifth, we did not perform head-to-head comparisons among different medications in the current study. At last, these trials reported continuous outcome metrics in various forms (*ie*, median, least-square mean difference, and ratio of adjusted geometric mean difference) without raw data, such as mean and standard deviation. As a result, the statistical approaches differ, and individual trial outcomes (significant or insignificant) may differ once transformed to SMD for this meta-analysis. Nonetheless, the pooled estimate remains meaningful, as it effectively consolidates the available evidence, despite possible inaccuracies. Future network meta-analyses will be needed to further understand the safety and efficacy of other emerging medications, including patisiran, inotersen, acoramidis, vutrisiran, and NI006, when additional literature is available.

## Conclusions

This meta-analysis, which included 2,973 patients, demonstrated that tafamidis treatment effectively improves clinical outcomes, slows amyloid accumulation, and halts the progression of myocardial remodeling. Our findings support tafamidis as a promising treatment for patients with ATTR-CM. However, the study also highlights the current inconsistency in protocols for monitoring treatment response. Standardizing these protocols remains a critical unmet clinical need for future research.

Perspectives **COMPETENCY IN MEDICAL KNOWLEDGE:** Tafamidis has been well-established in previous clinical trials as a pivotal disease-modifying medication for ATTR-CM. However, there is limited information on how tafamidis affects specific laboratory, functional, and imaging parameters. This study comprehensively demonstrates that tafamidis not only improves clinical outcomes but also reverses myocardial remodeling.

**TRANSLATIONAL OUTLOOK:** The long-term effects of tafamidis on pathophysiology and clinical progression warrant further investigation.

## Funding support and author disclosures

Dr Lin has received research grant support from 10.13039/100004319Pfizer. All other authors have reported that they have no relationships relevant to the contents of this paper to disclose.
