# Supplementary material for: Effect of Tafamidis on Clinical and Functional Parameters in Transthyretin Amyloid Cardiomyopathy
Source: JACC Adv. 2024 Dec 19;4(2):101511. doi: 10.1016/j.jacadv.2024.101511 (PMC11905165; doi:10.1016/j.jacadv.2024.101511)
Supplement: Supplemental Tables and Figures [file mmc1.docx]

**Supplemental Table 1. Search strategy for study inclusion**

| Database | Search syntax |
| --- | --- |
| PubMed | (“ATTR” OR “transthyretin”) AND (“cardiac”) AND (“amyloidosis”) AND (“echocardiography” OR “magnetic resonance imaging” OR “bone scintigraphy” OR “99mTechnetium‐pyrophosphate”) AND (“randomized controlled trial” OR “clinical trial” OR “cohort study” OR “case series” OR “case report”) |
| EMBASE | ('amyloidosis'/exp OR amyloidosis) AND (attr OR 'transthyretin'/exp OR transthyretin) AND ('cardiac'/exp OR cardiac) AND ('echocardiography'/exp OR 'echocardiography' OR 'magnetic resonance imaging'/exp OR 'magnetic resonance imaging' OR 'bone scintigraphy'/exp OR 'bone scintigraphy' OR '99mtechnetium‐pyrophosphate') AND ('randomized controlled trial':ab,ti OR 'clinical trial':ab,ti OR 'cohort study':ab,ti OR 'case series':ab,ti OR 'case report':ab,ti) |
| Cochrane’s library | (“ATTR” OR “transthyretin”) AND (“cardiac”) AND (“amyloidosis”) AND (“echocardiography” OR “magnetic resonance imaging” OR “bone scintigraphy” OR “99mTechnetium‐pyrophosphate”) AND (“randomized controlled trial” OR “clinical trial” OR “cohort study” OR “case series” OR “case report”) |

**Supplemental Table 2.** Cohort studies and randomized controlled trials were respectively appraised by ROBINS-I (Risk Of Bias In Non-randomized Studies - of Interventions) and Cochrane Risk of Bias

ROBINS-I for cohort studies

| Study | Confounding | Selection | Measurement of intervention | Missing data | Measurement of outcomes | Reported results |
| --- | --- | --- | --- | --- | --- | --- |
| Falk, 2011 | Moderate | Moderate | Low | Low | Moderate | Low |
| Fontana, 2021 | Moderate | Moderate | Low | Low | Moderate | Low |
| Doumas, 2022 | Serious | Serious | Low | Low | Serious | Low |
| Elsadany, 2022 | Serious | Serious | Low | Low | Serious | Low |
| Giblin, 2022 | Moderate | Moderate | Low | Low | Moderate | Low |
| Odouard, 2022 | Moderate | Moderate | Low | Low | Serious | Low |
| Rettl, 2022 | Moderate | Moderate | Low | Low | Moderate | Low |
| Chamling, 2023 | Serious | Moderate | Low | Low | Moderate | Low |
| Ghoneem, 2023 | Serious | Serious | Low | Low | Serious | Low |
| Gustafsson, 2023 | Serious | Serious | Low | Low | Serious | Low |
| Ichikawa, 2023 | Moderate | Moderate | Low | Low | Moderate | Low |
| Kim, 2023 | Moderate | Low | Low | Low | Moderate | Low |
| Lee, 2023 | Serious | Serious | Low | Low | Series | Low |
| Nakaya, 2023 | Serious | Serious | Low | Low | Series | Low |
| Papathanasiou, 2023 | Serious | Moderate | Low | Low | Series | Low |
| Rettl, 2023 | Moderate | Low | Low | Low | Moderate | Low |
| Takashio, 2023 | Moderate | Moderate | Low | Moderate | Moderate | Low |
| Tsai, 2023 | Moderate | Moderate | Low | Low | Moderate | Low |
| Wu, 2023 | Moderate | Moderate | Low | Low | Moderate | Low |
| Yu, 2023 | Moderate | Moderate | Low | Low | Moderate | Low |

Cochrane Risk of Bias for randomized controlled trial

| Study | Sequence generation | Allocation concealment | Blinding | Incomplete results | Selective reporting | Other bias |
| --- | --- | --- | --- | --- | --- | --- |
| Merlini, 2013 | Serious | Serious | Serious | Low | Moderate | Serious |
| Maurer, 2018 | Low | Low | Low | Low | Moderate | Low |
| Solomon, 2019 | Serious | Low | Low | Serious | Moderate | Moderate |
| Garcia-Pavia, 2023 | Moderate | Low | Low | Serious | Moderate | Low |
| Maurer, 2023 | Serious | Low | Low | Serious | Moderate | Moderate |
| Rosenblum, 2023 | Serious | Low | Low | Serious | Moderate | Moderate |
| Shah, 2023 | Low | Low | Low | Low | Moderate | Low |

**Supplemental Table 3.** Subgroup analysis of follow-up duration (≤12M vs. >12M)

| Outcome/ follow-up duration | No. of studies | OR / SMD  (95% CI) | *P* for subgroup difference |
| --- | --- | --- | --- |
| All-cause death |  |  | 0.891 |
| ≤12M | 3 | 0.17 (0.02, 1.50) |  |
| >12M | 2 | 0.21 (0.03, 1.56) |  |
| CV death |  |  | 0.057 |
| ≤12M | 1 | 0.04 (0.02, 0.11) |  |
| >12M | 1 | 0.17 (0.06, 0.47) |  |
| HHF |  |  | 0.244 |
| ≤12M | 3 | 0.34 (0.10, 1.19) |  |
| >12M | 1 | 0.84 (0.35, 2.00) |  |
| NTproBNP |  |  | 0.725 |
| ≤12M | 2 | -0.25 (-0.60, 0.10) |  |
| >12M | 1 | -0.18 (-0.37, 0.01) |  |

Abbreviation: CV, cardiovascular; HHF, heart failure hospitalization; OR, odds ratio; SMD, standardized mean difference; CI, confidence interval.

**Supplemental Figure 1.** Other echocardiographic parameters of studies which pooled the data of tafamidis and placebo separately, including (A) E' lateral; (B) E’ septal; (C) IVSD; (D) LVEDD; (E) LVESD; (F) LVPWD. Abbreviations: IVSD: interventricular septal distance; LVEDD: left ventricular end-diastolic diameter; LVESD: left ventricular end-systolic diameter; LVPWD: left ventricular postural wall thickness.


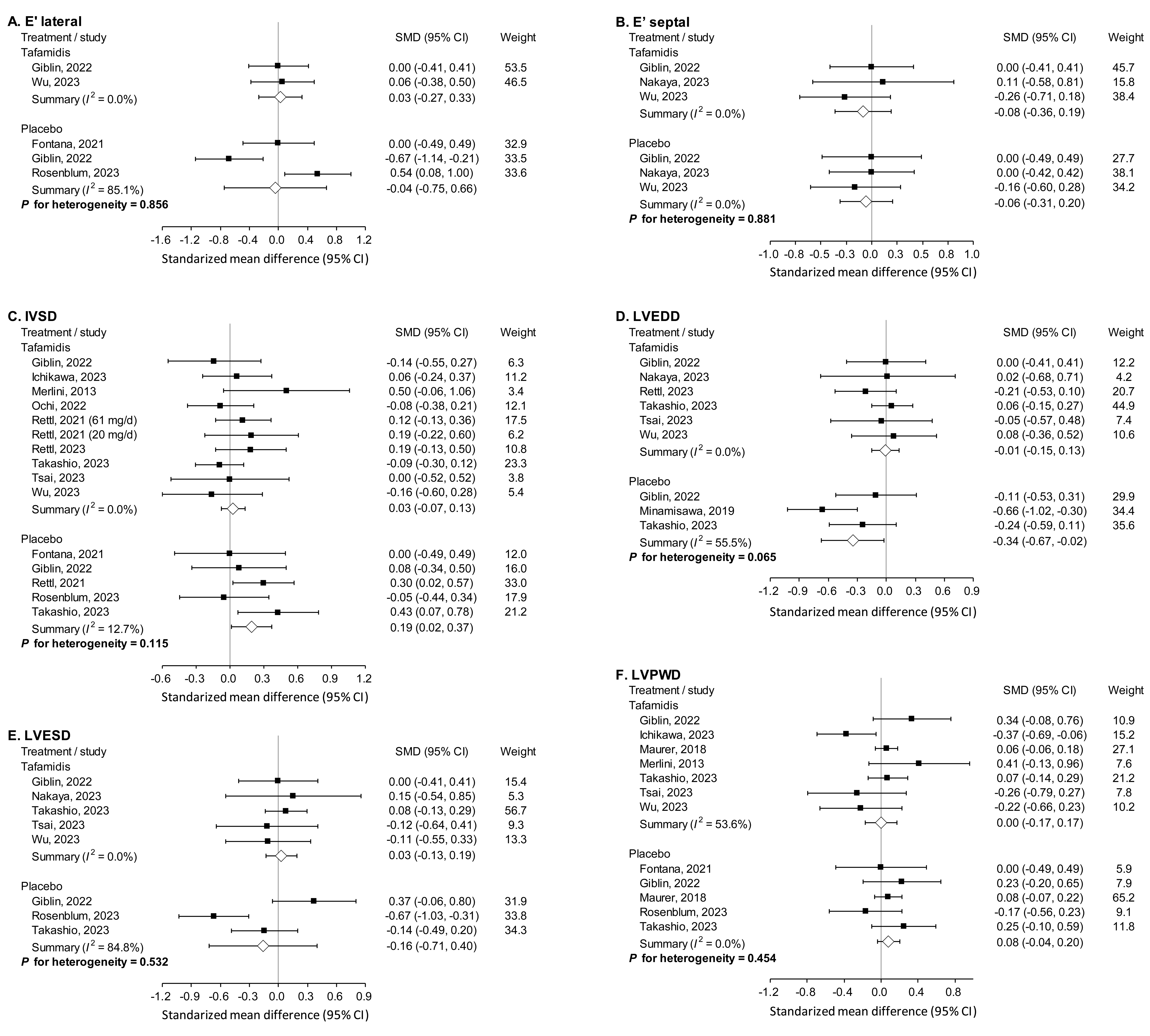


**Supplemental Figure 2.** Other speckle tracking parameters of studies which pooled the data of tafamidis and placebo separately, including (A) 2 chamber LS; (B) 3 chamber LS; (C) 4 chamber LS. Abbreviations: LS: longitudinal strain.





**Supplemental Figure 3.** Other CMR parameters of studies which pooled the data of tafamidis and placebo separately, including (A) IVS; (B) LA empty fraction; (C) LALS; (D) LVCI; (E) LVSVI; (F) LVEDVI; (G) RVCI; (H) RVSVI; (I) RVEDVI. Abbreviations: CMR: cardiac magnetic resonance imaging; IVS: inter-ventricular septum; LALS: left atrial longitudinal strain; LVCI: cardiac index; LVSVI: left ventricular stroke volume index; LVEDVI: left ventricular end-diastolic volume index; RVCI: right ventricular cardiac index; RVSVI: right ventricular stroke volume index; RVEDVI: right ventricular end-diastolic volume index.


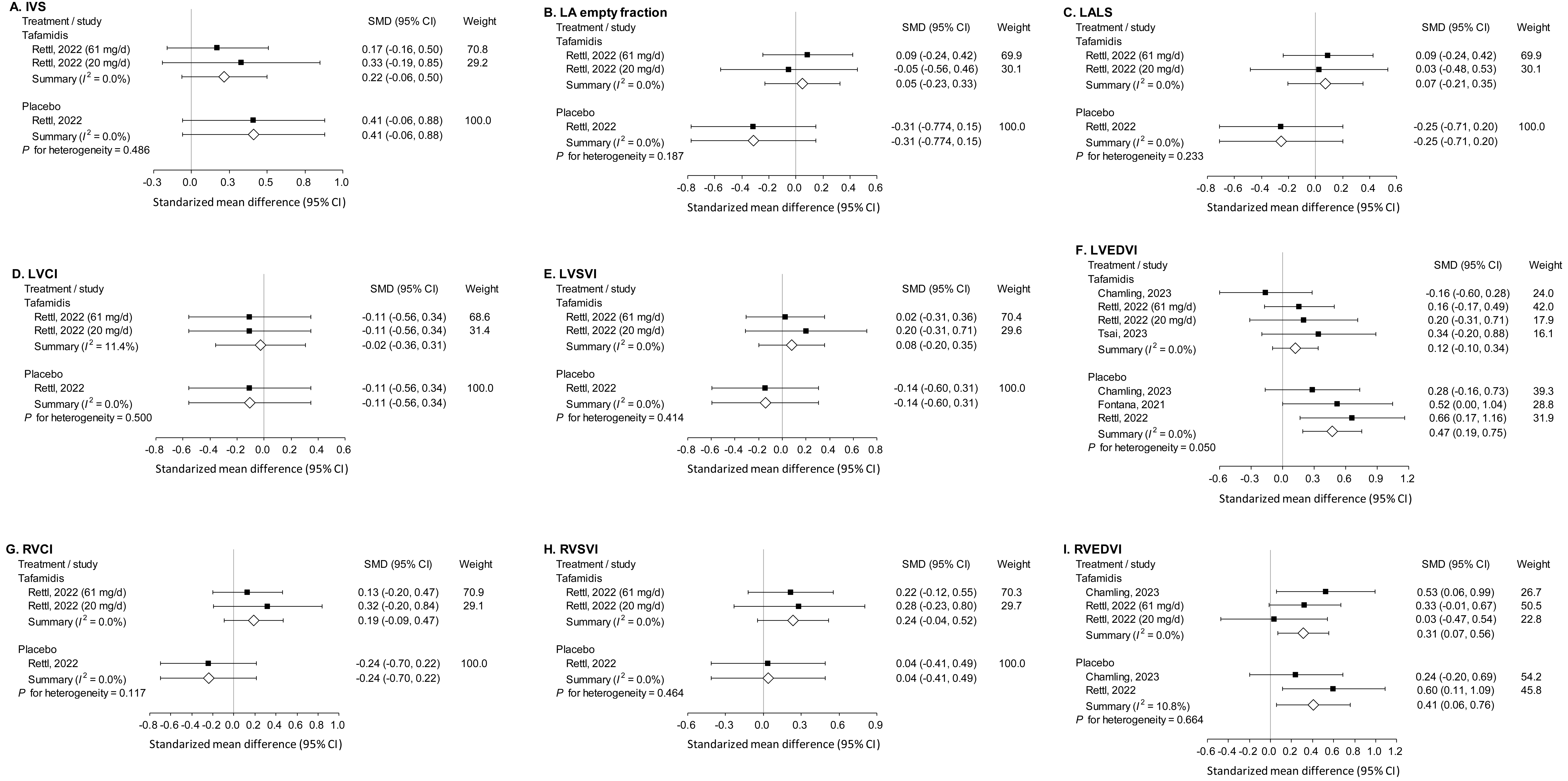


**Supplemental Figure 4.** Subgroup analysis on all-cause mortality and NT-proBNP to compare results between analyses that included and excluded randomized controlled trials (RCT). Abbreviation: NT-proBNP: N terminal pro B type natriuretic peptide.
